# Changes in Glomerular Filtration Rate After Switching From Tenofovir Disoproxil Fumarate to Tenofovir Alafenamide Fumarate for Human Immunodeficiency Virus Preexposure Prophylaxis

**DOI:** 10.1093/ofid/ofad695

**Published:** 2023-12-29

**Authors:** Adovich S Rivera, Katherine Pak, Matthew T Mefford, Rulin C Hechter

**Affiliations:** Department of Research and Evaluation, Kaiser Permanente Southern California, Pasadena, California, USA; Department of Research and Evaluation, Kaiser Permanente Southern California, Pasadena, California, USA; Department of Research and Evaluation, Kaiser Permanente Southern California, Pasadena, California, USA; Department of Research and Evaluation, Kaiser Permanente Southern California, Pasadena, California, USA; Department of Health Systems Science, Kaiser Permanente Bernard J. Tyson School of Medicine, Pasadena, California, USA

**Keywords:** HIV, preexposure prophylaxis, renal function, tenofovir alafenamide

## Abstract

**Background:**

Tenofovir alafenamide fumarate (TAF) was promoted as a safer alternative to tenofovir disoproxil fumarate (TDF) for human immunodeficiency virus oral preexposure prophylaxis (PrEP). It is unknown if switching from TDF to TAF translates to improved renal function. We used electronic health record (EHR) data to assess changes in creatinine-estimated glomerular filtration rate (eGFR) after switching from TDF to TAF.

**Methods:**

We conducted a retrospective cohort study using EHR data from Kaiser Permanente Southern California. We identified individuals who switched from TDF to TAF between October 2019 and May 2022 and used time-varying propensity score matching to identify controls who were on TDF (“nonswitchers”). We then used Bayesian longitudinal modeling to compare differences in eGFR between switching and nonswitching scenarios.

**Results:**

Among 5246 eligible individuals, we included 118 TDF to TAF switchers and 114 nonswitchers. Compared to nonswitchers, switchers had older age of starting TDF but similar body weights at index date. A higher proportion of switchers were White, on Medicare or Medicaid, and had dyslipidemia at index date. Switching to TAF was associated with a higher eGFR compared to staying on TDF in 3–15 months post-switch, but the differences were not statistically significant (eg, month 9 difference: 1.27 [95% credible interval, −1.35 to 3.89]). While most of the estimated changes showed eGFR increase associated with switching, most were <2 eGFR units. Sensitivity analyses to address missingness or nonadherence showed similar results.

**Conclusions:**

Switching from TDF to TAF for PrEP was associated with a nonsignificant increase in eGFR. Findings need to be confirmed using larger cohorts.

## Teaser

Using data from an integrated health system in California, we found there was a nonsignificant increase in estimated glomerular filtration rate associated with switching from tenofovir disoproxil fumarate to tenofovir alafenamide fumarate for HIV preexposure prophylaxis.

In October 2019, oral, once-daily emtricitabine/tenofovir alafenamide fumarate (TAF) was approved for human immunodeficiency virus (HIV) preexposure prophylaxis (PrEP) by the US Food and Drug Administration (FDA) [1]. TAF was promoted as having a better safety profile for renal toxicity compared to the older PrEP regimen approved in 2012 containing tenofovir disoproxil fumarate (TDF) [1–3]. The Study to Evaluate the Safety and Efficacy of Emtricitabine and Tenofovir Alafenamide Fixed-Dose Combination Once Daily for Pre-Exposure Prophylaxis in Men and Transgender Women Who Have Sex With Men and Are At Risk of HIV-1 Infection (DISCOVER) trial showed that those assigned to TAF exhibited more favorable long-term renal function trajectories (ie, lower reductions in estimated glomerular filtration rate [eGFR] over time) compared to those on TDF [4]. Pooled analysis of data from 12 PrEP trials also found increased adverse renal events (eg, mild asymptomatic elevation in eGFR) in people on TDF versus placebo [5]. TAF is hypothesized to be safer for renal function as it leads to lower plasma levels of tenofovir, translating to lower nephrotoxicity [6, 7]. Currently, FDA labels show that TAF, but not TDF, can be used by people who have renal clearance <60 mL/minute, which is indicative of kidney dysfunction [8, 9].

Some people who initiated PrEP with TDF have started switching to TAF, citing convenience and safety reasons such as small tablet size, side effects, and vulnerability to renal function decline due to preexisting conditions [3, 10]. Data from people with HIV (PWH) support switching for renal safety reasons. Pooled analysis of trials in people with HIV showed that switching from TDF to TAF-based regimens led to positive changes in renal biomarkers (eg, creatinine clearance, urinary biomarkers) compared to staying on TDF [11]. Similarly, a cohort study found that PWH who switched from TDF to TAF experienced improvement in eGFR [12]. Results from DISCOVER, which compared TDF to TAF for PrEP, also supports switching. They found that TDF-experienced individuals assigned to the TAF arm (essentially, leading to switching) had higher eGFR on 48-week follow-up than TDF-experienced individuals assigned to the TDF arm, as eGFR increased in the TAF arm while remaining stable in the TDF arm [13]. Results from the open-label phase of DISCOVER also showed that PrEP users switching from TDF to TAF exhibited increase in eGFR (relative to baseline), while those who stayed on TAF had a relative decline [14]. However, the DISCOVER study population does not necessarily reflect the demographic characteristics of people on PrEP in the US [15, 16], and it would be important to confirm findings in nontrial settings. In this retrospective cohort study using electronic health records (EHRs), we assessed changes in eGFR after switch from TDF to TAF in a matched cohort study where we compare TDF-to-TAF switchers to those who stayed on TDF.

## PATIENTS AND METHODS

### Study Design

This is a retrospective cohort study using EHR from Kaiser Permanente Southern California (KPSC). KPSC is an integrated healthcare delivery system providing comprehensive health services to approximately 4.8 million members. KPSC membership is diverse and widely representative of the communities in the Southern California service area, comprising >20% of Southern California's population [17]. Members’ receipt of outpatient, inpatient, laboratory, and pharmacy services are tracked in KPSC's EHR system. Care received out of KPSC facilities is captured through billing claims.

### Study Population

Adults ≥18 years of age who initiated PrEP at KPSC with TDF during 2014 to 2021 were identified for the underlying study cohort. Individuals were considered eligible for regimen switching and entered the cohort on their switch-eligible date. This is the date when they have used TDF for PrEP for at least 6 months (based on pills received) or 1 October 2019, whichever came later. Individuals were excluded if they disenrolled or initiated TAF with <6 months of TDF use. We also excluded individuals who had chronic kidney disease or abnormal laboratory findings (eg, grade 3 or 4 proteinuria) at time of TDF initiation (see Supplementary Methods for full details).

This underlying cohort was then used to identify matched TDF-TAF switchers and TDF-only nonswitchers via time-varying propensity score (TV-PS) matching. Details on matching and calculation of the assigned switch date are described in the statistical analysis section.

### Exposure, Outcome, and Follow-up

The exposure of interest is switching from TDF to TAF after cohort entry and was based on pharmacy filled (sold) data. Switchers were compared to those who stayed on TDF (ie, nonswitchers).

The outcome is creatinine-based eGFR (henceforth, “eGFR”) after switching to TAF up to 18 months of follow-up or study end date (30 June 2022). All eGFRs were recalculated using the Chronic Kidney Disease Epidemiology Collaboration (CKD-EPI) 2021 creatinine equation [18]. The index date for follow-up started on the recorded switch date for switchers and the assigned switch date for nonswitchers.

### Covariates

Covariates included age at TDF initiation, sex, race and ethnicity (Asian [non-Hispanic], Black [non-Hispanic], Hispanic, White [non-Hispanic], other), insurance type (commercial, Medicaid/care, others), ever smoking, cardiometabolic comorbidities (diabetes, dyslipidemia, hypertension; time-varying), and body weight (time-varying). Comorbidities were identified using combinations of diagnosis codes, medication, and laboratory data (see Supplementary Methods for definitions). Missing covariates were handled using single imputation with random forests for computation efficiency [19].

### Statistical Analysis: Matching

TV-PS matching was performed to identify matched switchers and nonswitchers following the approach described by Zhang et al [20]. This achieves some covariate balance but more importantly, allowed the identification of an index date to start follow-up in nonswitchers. The index date for switchers would be their actual switch date.

**Figure 1.**
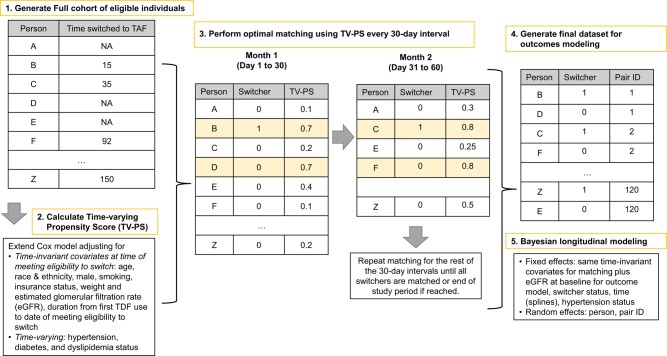
Creation of matched sample. Numbers in the figure are illustrative and do not represent the actual observed or calculated values. Abbreviations: eGFR, estimated glomerular filtration rate; NA, not available; TAF, tenofovir alafenamide fumarate; TDF, tenofovir disoproxil fumarate; TV-PS, time-varying propensity score.

The TV-PS model was an extended Cox model that included time-invariant (age, race and ethnicity, male, ever smoking, insurance, year of switch eligibility met, weight, and eGFR at switch-eligible date) and time-varying (diabetes, dyslipidemia, and hypertension status) covariates. An iterative process (Figure 1) was then used to perform 1:4 matching without replacement. Since matching is done in the order of switch dates, individuals were eligible to be selected as a matched nonswitcher prior to their TDF-TAF switch date.

After matching, an index date was assigned for nonswitchers by calculating the interval between the switcher's switch-eligible date and actual switch date and adding this number of days to the nonswitchers’ switch-eligible date [20, 21]. Therefore, the index date of switchers and nonswitchers would not necessarily be the same calendar date, but both groups will have similar distribution of intervals between the switch eligibility date and the follow-up index date.

### Statistical Analysis: Outcome Analysis

Using the matched cohort, we first calculated descriptive statistics for clinical-demographic variables to compare switchers to matched nonswitchers and, separately, compared the matched cohort to the original group of eligible individuals. We also plotted eGFR over time from the follow-up index date to month 18 post–index date.

We used Bayesian linear mixed effects models to examine eGFR under the counterfactual scenario that TDF-to-TAF switchers did not switch their regimen. In other words, we estimated “What would have happened to the eGFR if the switchers in the data did not switch?” given the switchers’ covariate distribution. This is equivalent to estimating average treatment effect among the treated [22]. We calculated differences in eGFR between switching and nonswitching scenarios at a priori selected time points after switching (months 0.5, 3, 6, 9, 12, 15, and 18). In this main analysis, individuals with no follow-up eGFR data after the (assigned) switch date were further excluded.

The Bayesian model included spline terms for time and were adjusted for age, sex, insurance, race and ethnicity, ever smoking, eGFR at index date, and weight at index date. Random effects for person and pair and interaction terms (eg, time × switch status) were also included. Bayesian models produce 95% credible intervals (CrIs) instead of confidence intervals. The 95% CrI is interpreted as “there is a 95% probability that the true effect estimate would lie within the interval, given the evidence provided by the observed data” [23]. We calculated 2 additional measures to aid interpretation: probability of direction (PDI) and region of practical equivalence (ROPE). PDI tells us the proportion of the (posterior) estimate lies in the direction of positive difference (ie, switching is favorable) in eGFR [24]. ROPE tells us the percentage of the posterior draws that lie in the region of “practically no effect or difference,” which we set at ±2 eGFR units [25]. We performed 3 sensitivity analyses. First, we repeated modeling but limited it to eGFR during the time that individuals were adherent with their exposure at index date (eg, nonswitchers remained on TDF). This partially handles the issue of nonswitchers including people who were observed to switch later during follow-up. The second and third sensitivity analyses were conducted to address the issue of missing eGFR. In the second sensitivity analysis, we used a weighted Bayesian model incorporating inverse probability missingness weights [26]. Stabilized weights were calculated from logistic regression models on the probability of being included in the complete case analysis. In the third sensitivity analysis, missing outcomes and covariates were imputed using multiple imputation with chained equations and the models were run using separate linear regression models (1 for each time point) instead of using a mixed effects model.

All analyses were conducted in R/RStudio version 4.3.0. Technical details of the modeling approaches and the sample code are available in the Supplementary Methods.

## RESULTS

We identified 5246 eligible individuals who initiated TDF in the TV-PS matching (Supplementary Figure 1). At switch-eligible date, these individuals had a mean age of 38 years (standard deviation [SD], 11 years), 98% were male, 35% were Hispanic, 40% were non-Hispanic White, 80% had commercial insurance, and 25% had diabetes.

Our final analytical dataset for eGFR modeling included 528 (118 switchers and 410 matched nonswitchers). There were 125 individuals who switched from TDF to TAF during follow-up, (Supplementary Table 1), among which 118 TDF to TAF switchers were matched to 410 nonswitchers who had at least 1 measured eGFR value post-switch (Supplementary Figure 1). Latest included switch date was in May 2022. Seven of the initial 125 switchers were selected as a matched nonswitcher prior to their switch and were treated as nonswitchers for the rest of the analysis. Compared to nonswitchers, switchers were older at TDF initiation (45 [SD, 13] years vs 38 [SD, 10] years) but had similar baseline body weight (85 [SD, 14] kg vs 84 [SD, 16] kg). A higher proportion of switchers were White (50% vs 44%), on Medicare or Medicaid (12% vs 4%), and had dyslipidemia (2% vs 1%) at index date. Switchers also had a longer time of using TDF based on time from first TDF dispense date to meeting the switch eligibility criteria, and time from first TDF dispense date to index date for eGFR modeling (Table 1).

**Table 1.** Descriptive Statistics of Switchers and Matched Nonswitchers, Kaiser Permanente Southern California, October 2019–June 2022

Baseline Variable	Switcher (n = 118)	Nonswitcher (n = 410)
Age, y, mean (SD)	44.86 (12.80)	38.00 (10.62)
Time to meeting switch eligibility, d, median (IQR)	534 (250–961)	363 (229–879)
Time from first TDF to index date for eGFR modeling, d, median (IQR)	806 (484–1414)	742 (392–1221)
Weight, kg, mean (SD)	84.19 (17.09)	85.41 (19.49)
eGFR^a^, mL/kg/m^2^, mean (SD)	86.07 (20.93)	99.44 (16.27)
Race and ethnicity^b^		
Asian, non-Hispanic	13 (11.0)	47 (11.5)
Black, non-Hispanic	7 (5.9)	16 (3.9)
Hispanic	29 (24.6)	124 (30.2)
White, non-Hispanic	59 (50.0)	180 (43.9)
Other, non-Hispanic	10 (8.5)	43 (10.5)
Male	117 (99.2)	399 (97.3)
Insurance type		
Commercial	81 (68.6)	342 (83.4)
Medicaid	9 (7.6)	17 (4.1)
Medicare	5 (4.2)	2 (0.5)
Other	23 (19.5)	49 (12.0)
Ever smoked	33 (28.0)	99 (24.1)
Dyslipidemia	2 (1.7)	3 (0.7)
Hypertension	3 (2.5)	10 (2.4)

Data are presented as No. (%) unless otherwise indicated.

Abbreviations: eGFR, estimated glomerular filtration rate; IQR, interquartile range; SD, standard deviation; TDF, tenofovir disoproxil fumarate.

^a^Calculated using the Chronic Kidney Disease Epidemiology Collaboration 2021 creatinine equation.

^b^Other race and ethnicity includes multiracial, Native American/Alaska Native, Pacific Islander, and all other types of responses not reported in the table.

### Estimated Glomerular Filtration Rate Over Time

**Figure 2.**
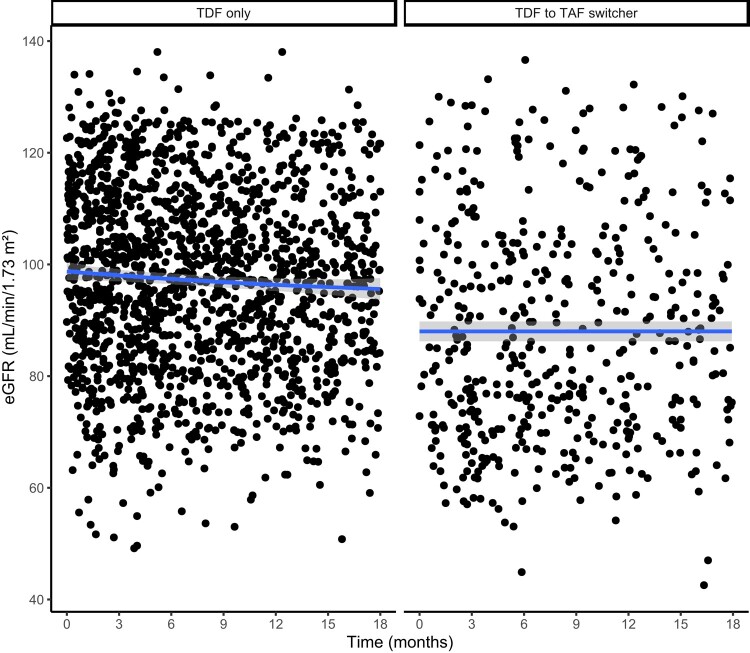
Observed estimated glomerular filtration rate (eGFR) over time in matched sample, Kaiser Permanente Southern California, October 2019–June 2022. Time 0 is switch date for tenofovir disoproxil fumarate (TDF) to tenofovir alafenamide fumarate (TAF) switchers and assigned switch date for TDF-only nonswitchers. Trend line was fit using the *geom_smooth* function. Estimated eGFR was calculated using the Chronic Kidney Disease Epidemiology Collaboration 2021 creatinine equation.

Switchers had significantly lower baseline (ie, at index date) eGFR (86 [SD, 21] vs 100 [SD, 16] mL/minute/1.73 m^2^) compared to nonswitchers. Over time, average eGFR remained generally lower among switchers compared to nonswitchers across all assessment time points up to the end of the study period (Supplementary Figure 2). For example, switchers had a lower median eGFR than matched nonswitchers at month 3 (85 vs 98) and month 18 (88 vs 97). The data were widely scattered, but the linear smoothing function suggested a downward trend of eGFR over time that is more noticeable in the nonswitcher group (Figure 2).

### Estimated Glomerular Filtration Rate After Switching

**Figure 3.**
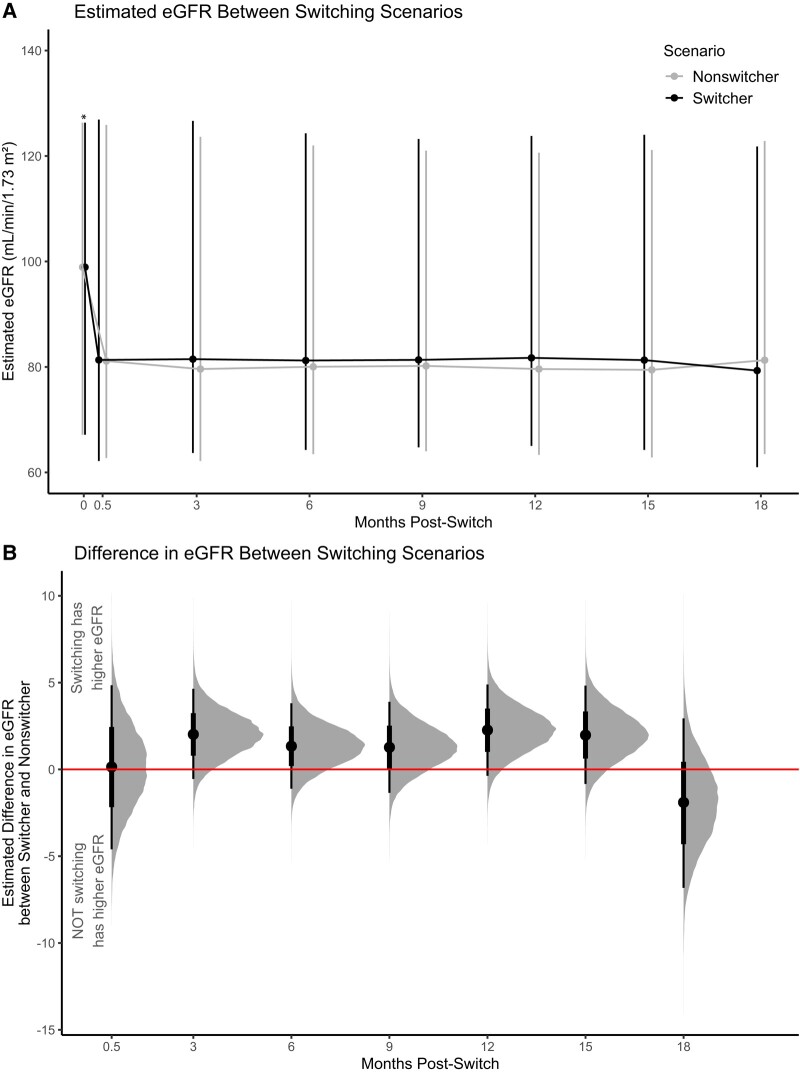
Estimated difference in estimated glomerular filtration rate (eGFR) between switching and nonswitching scenarios, Kaiser Permanente Southern California, October 2019–June 2022. eGFR was based on the Chronic Kidney Disease Epidemiology Collaboration 2021 creatinine equation. Estimates were from a Bayesian mixed effect model. Fixed effects include switch status, age, male gender, insurance status, race and ethnicity, smoking, weight, and eGFR at switch-eligible date, duration from starting TDF to switch-eligible date, hypertension status at baseline, eGFR at baseline, time (splines). Random effects include: person, pair. Pairwise interaction terms for switch status, eGFR at baseline, and time also included. All estimates were based on baseline covariate of TDF to TAF switchers. In *A*, dot represents point estimate while vertical segments represent 95% credible intervals (CrIs). In *B*, differences represent average treatment effect among the treated. Gray density curve shows distribution of estimated differences. Dot is median of estimated differences. Thick lines show 66% CrI and thin lines show 95% CrI. Horizontal line through zero is threshold for no difference.

Switching to TAF was associated with a higher eGFR compared to staying on TDF in 3–15 months post-switch, but 95% CrIs crossed the null and were not statistically significant (Figure 3). Month 12 data showed the greatest gap (2.26 [95% CrI, −.37 to 4.89]) while month 18 data showed a reversal of difference (−1.90 [95% CrI, −6.82 to 2.94]) (Table 2). While the findings are null, the PDI showed that most (>85%) draws of estimated differences suggest elevated eGFR from month 3 to month 15 if switching occurred (Supplementary Table 2). Despite this high probability that switching translated to eGFR, ROPE suggests that differences were practically (clinically) equivalent to no difference as around half of points are within the regions of no meaningful difference (±2 units change in eGFR) (Supplementary Table 2). The findings remained similar after censoring eGFR values measured after treatment deviation (eg, TAF switcher switched back to TDF) (Supplementary Figure 3).

**Table 2.** Estimated Difference in Estimated Glomerular Filtration Rate Between Switching and Nonswitching Scenarios, Kaiser Permanente Southern California, October 2019–June 2022

Month Post–Index Date	Estimated Difference^a^ in eGFR^b^			
	Main Analysis (95% CrI)	SA1 (95% CrI)	SA2 (95% CrI)	SA3 (95% CI)
0.5	0.14 (−4.6 to 4.85)	0.81 (−4.21 to 5.59)	0.03 (−4.6 to 4.6)	1.27 (−1.05 to 3.58)
3	2.02 (−.55 to 4.64)	1.95 (−.73 to 4.67)	2.16 (−.44 to 4.70)	0.88 (−1.44 to 3.21)
6	1.34 (−1.11 to 3.81)	1.33 (−1.25 to 3.93)	1.26 (−1.20 to 3.70)	1.03 (−1.14 to 3.19)
9	1.27 (−1.35 to 3.89)	1.07 (−1.68 to 3.81)	1.08 (−1.55 to 3.65)	−1.15 (−3.7 to 1.4)
12	2.26 (−.37 to 4.89)	1.68 (−1.15 to 4.52)	2.21 (−.43 to 4.77)	2.94 (.39–5.49)
15	1.98 (−.84 to 4.82)	2.31 (−.80 to 5.41)	2.11 (−.72 to 4.86)	2.82 (−.13 to 5.77)
18	−1.90 (−6.82 to 2.94)	2.08 (−4.15 to 8.54)	−1.86 (−6.60 to 2.91)	2.82 (−.13 to 5.77)

After modeling, estimated eGFR of 2 scenarios using covariate data of switchers only was calculated. The difference of the 2 scenarios is shown here and corresponds to an estimate of the average treatment effect among the treated. Main analysis used Bayesian mixed effects longitudinal model. SA1 used the same model but limited the data up to time where person was adherent to their switch status at baseline (eg, eGFR after nonswitcher starts TDF was excluded). SA2 used the same Bayesian models but weighted with inverse propensity missingness weights. SA3 used linear regression with multiple imputation.

Abbreviations: CI, confidence interval; CrI, credible interval; eGFR, estimated glomerular filtration rate.

^a^Difference = estimated eGFR in switching scenario – estimated eGFR in nonswitching scenario.

^b^Calculated using the Chronic Kidney Disease Epidemiology Collaboration 2021 creatinine equation.

The sensitivity analyses incorporating missingness weights (SA2) or using multiple imputation (SA3) showed similar results, although the month 12 difference with multiple imputation was found to be significant (2.94 [95% confidence interval, .39–5.49]) (Table 2, Supplementary Figures 3 and 4).

## DISCUSSION

We found that switching from TDF to TAF was not associated with increased eGFR among adults on PrEP. The difference in the short-term eGFR trajectory was not statistically or clinically significant based on credible intervals and ROPE results. Our results do not support that switching from TDF to TAF would improve eGFR in our sample of insured patients using PrEP. This contrasts with the findings of DISCOVER where TDF-experienced individuals assigned to the TAF arm (ie, switched to TAF) had significantly higher eGFR by week 48 compared to those assigned to TDF [13].

The main proposed renal protective mechanism of TAF relates to the lower tenofovir plasma levels, which lead to lower injury of the proximal tubule cells [27]. Lower tenofovir plasma levels due to switching may have allowed recovery of epithelial cells and improvements in eGFR as observed in DISCOVER [13]. The null results in our analysis could be due to differences in our population, such as age and eGFR at index dates, suggesting the possibility of heterogenous impact of switching by these variables. This highlights the value of examining outcomes when the target patient population differs from the trial population, especially if the differences influence the outcome [28]. There could be individuals who have already had irreversible damage from TDF use such that switching would have little impact, echoing observations in TDF discontinuation studies in PWH [29, 30]. The null findings could also be due to unmeasured confounding. For example, we were not able to fully account for cumulative TDF exposure prior to switch or for PrEP adherence or off-label intermittent use of PrEP [31, 32]. Finally, tenofovir from TAF remains excreted in the urine so the proximal tubule remains exposed, albeit at lower levels [33].

Our article also serves as a practical demonstration on how to study the impact of switching treatments using real-world data through matching [20, 34]. A common approach would be to compare outcomes of switchers before and after switching [35]. However, this approach lacks a control group. Another approach would be to compare ever switchers to never switchers [36, 37]. The first issue with this is that it can create groups that cannot be made comparable at baseline even with covariate adjustment. Never switchers likely have some indication that prevent them from using the alternative strategy (also known as positivity violation in epidemiology) [38]. In the setting of survival analysis, comparing ever and never switchers can introduce immortal time bias, as outcomes prior to switching are always counted in the never switcher group (ie, ever switchers are made immune to the outcome prior to switching) [39]. For our analysis, we focused on continuous outcomes so the main challenge is identifying the appropriate index date to start follow-up. Importantly, even if we just compare never to ever switchers, we will still not have an appropriate index date for follow-up. While one could theoretically assign a random date to start follow-up among never switchers, this does not fully address immortal time bias. We overcome these challenges by using time-varying propensity scores combined with outcome modeling [20]. The approach allowed us to select a contemporary control without conditioning on future events. Alternative approaches include sequential nested trials and marginal structural models, but these methods work better in settings with larger samples and more regular timing of assessments [40, 41].

This study has some limitations. First, missing data, especially clinical or laboratory data, was an issue. We used single imputation for some missing covariate data for computation efficiency. However, studies have shown that multiple imputation is the gold standard [42]. Relatedly, we limited our analysis to eGFR, as the more specific measures of renal tubular proteinuria (eg, urine β_2_-microglobulin to creatinine ratio, retinol-binding protein to creatinine ratio) cannot be obtained from our EHR data [13, 43]. While measures for microalbuminuria (eg, albumin-to-creatinine ratio) are also available, these are not routinely measured on follow-up for PrEP in our system. Second, our TV-PS model does not fully account for important TV variables that affect decision to switch like eGFR or lipids due to missingness. Instead, we incorporated these variables in the outcome model. However, this leads to our results being interpretable only as average treatment effect among the treated [22]. We cannot make inference on the counterfactual for controls (ie, what would happen to eGFR of nonswitchers had they switched to TAF?). The approach we adapted also does not consider replacement of potential matches, which requires modified analytic procedures [20, 22]. Alternative techniques can be used to overcome these issues, such as a sequence of target trials, but that requires a large sample of switchers and, ideally, more regularly measured time-varying covariates [41]. Third, our study was also limited to short follow-up since TAF was approved for PrEP use in 2019. Relatedly, the reversed direction at month 18 could be due to data sparsity since we observed that only around 28% have observed eGFR data by month 18. Fourth, we should ideally incorporate time-varying censoring weights to account for adherence [44], but it was not feasible to implement these more complicated models given available structured data in the EHR. Instead, we conducted a sensitivity analysis where we censored eGFR after nonadherence. We also assumed that individuals were following a once-daily PrEP regimen and not taking it intermittently or “on demand.” Fifth, we focused on renal function measured by eGFR rather than clinical events. Finally, since our data came from an integrated health system in Southern California, generalizability to other healthcare settings or uninsured or underinsured patient populations may be limited.

In summary, we found that switching from TDF to TAF was associated with a nonsignificant increase in eGFR among people on PrEP. Confirmatory studies with larger cohorts and longer follow-up or randomized switch or crossover trials are needed to confirm our findings.

## Supplementary Material

ofad695_Supplementary_Data

## Supplementary Data

Supplementary materials are available at *Open Forum Infectious Diseases* online. Consisting of data provided by the authors to benefit the reader, the posted materials are not copyedited and are the sole responsibility of the authors, so questions or comments should be addressed to the corresponding author.

## Notes

***Data availability.*** Data are not publicly available. Anonymized data that support the findings of this study may be made available from the investigative team in the following conditions: (1) agreement to collaborate with the study team on all publications, (2) provision of external funding for administrative and investigator time necessary for this collaboration, (3) demonstration that the external investigative team is qualified and has documented evidence of training for human subjects protections, and (4) agreement to abide by the terms outlined in data use agreements between institutions.

***Patient consent.*** The Kaiser Permanente Southern California institutional review board reviewed and approved the study protocol with a waiver of informed consent.
