# Supplementary material for: Changes in Glomerular Filtration Rate After Switching From Tenofovir Disoproxil Fumarate to Tenofovir Alafenamide Fumarate for Human Immunodeficiency Virus Preexposure Prophylaxis
Source: Open Forum Infect Dis. 2023 Dec 29;11(2):ofad695. doi: 10.1093/ofid/ofad695 (PMC10863550; doi:10.1093/ofid/ofad695)
Supplement: ofad695_Supplementary_Data [file ofad695_supplementary_data.zip › prep o2 - eGFR and TAF swtiching - SFile 1 - methods.docx]

**Supplement File 1:** **Supplemental Methods**

**Changes in Glomerular Filtration Rate after Switching from Tenofovir Disoproxil Fumarate to Tenofovir Alafenamide Fumarate for HIV Pre-exposure Prophylaxis**

Adovich S. Rivera, MD, PhD^1^

Katherine Pak, MS^1^

Matthew Mefford, PhD^1^

Rulin C. Hechter, MD, PhD^1.2^

^1^ – Department of Research and Evaluation, Kaiser Permanente Southern California

^2^ – Health Systems Science, Kaiser Permanente Bernard J. Tyson School of Medicine

**CONTENTS**

1. Operational definitions for Comorbidities
2. Laboratory tests in eligibility criteria
3. Single imputation of missing data for matching using random forest
4. Time-varying propensity score matching
5. Bayesian modeling
6. Sensitivity analysis: Compliance
7. Sensitivity analysis: Compliance
8. Sensitivity analysis: Weighted Bayesian model
9. References Cited
10. Sample R code
11. **Operational definitions for Comorbidities**

Dyslipidemia is defined as having At least 2 ICD codes (ICD9: 272, ICD10: E78.0, E78.00, E78.01, E78.1, E78.2, E78.3, E78.4, E78.41, E78.49, E78.5) for dyslipidemia within 2-year period or abnormal Lipid levels plus initiation of statin within a 6-month period.

Diabetes is defined as having (i) any one ICD code (ICD9: 250.x, ICD10: E10.x, E11.X, E13.X) AND (HbA1c >= 6.5% OR use of anti-diabetes medication) OR (ii) having at least two ICD codes for diabetes

Hypertension was ascertained using both ICD-10 diagnosis codes (I10, I15.xx) and outpatient blood pressure measurements, and defined as ≥2 diagnosis codes during separate encounters in any care setting or ≥2 abnormal outpatient blood pressure measurements (SBP ≥140 mmHg or diastolic blood pressure (DBP) ≥90mmHg) occurring on separate dates within a two-year period.

Chronic kidney disease was based on having ≥1 ICD code for Chronic Kidney disease (ICD9: 585, 585.1, 585.2, 585.3, 585.4, 585.5, 585.6, 585.9; ICD10: N18.1, N18.2, N18.3, N18.4, N18.5, N18.6, N18.9) OR has renal transplant OR on dialysis OR have 2 abnormal estimated glomerular filtration rate (eGFR) at least 3 months apart. Transplant and dialysis was based on internal renal disease registry data.

1. **Laboratory tests in eligibility criteria**

To be eligible for analysis, individuals should not have evidence of abnormal laboratory results at baseline. Abnormal labs are:

- Alanine transaminase ≥ 2.5 upper limit of normal (ULN) (ULN = 63)
- Aspartate transaminase ≥ 2.5 ULN (ULN = 34)
- Total bilirubin > 1.5 mg/dL
- Direct bilirubin < 0.3 mg/dL
- Absolute neutrophil count < 1000/mm^3^
- Platelets < 75/mm^3^
- Hemoglobin <10 g/dL
- Grade 3 or 4 glucosuria
- Grade 3 or 4 proteinuria

1. **Single imputation of missing data for matching using random forest**

Missing covariates needed for matching were handled using single imputation with random forests for computation efficiency using missForest.^1^ The model included age, race & ethnicity, gender, ever smoking, insurance type, index year, comorbidity status, time to meeting eligibility to switch, time to switch from switch eligibility date, and time to end of follow-up. It also included weight, body mass index and eGFR at time of switch eligibility and at time of TDF start. The algorithm used a maximum iteration of 10 and up to 100 trees for the forest.

1. **Time-varying propensity score matching**

Our approached followed the process described by Zhang et al.^2^ where we first generate the propensity score for switching then used an iterative and sequential process to generate the matched switchers and non-switchers without replacement.

Our propensity score model used a Cox proportional hazards model as implemented in the `survival` package.^3^ We regressed time-to-switch on time-fixed covariates: age, race, gender, ever smoking, insurance, cardiometabolic comorbidities, year of switch eligibility, weight at switch eligibility, eGFR at switch eligibility, and time from TDF start to switch eligibility. From that model, we generated the cumulative hazard of switching using the `survfit` function.

We then generated a person-time dataset which contains copies of a person’s data from time zero (switch eligibility date) up to day 1020 that is bracketed into 30-day intervals. We merged this data with the cumulative hazard and assigned the maximum cumulative hazard within interval as the propensity score of a person for that interval.

Finally, we proceeded with the matching using an optimal matching algorithm as implemented in the `optmatch` package.^4^ In each interval starting, we identified people who switched then used optmatch to find four matches from people who have not switched during that interval. The switchers and matched non-switchers are then removed from the pool before proceeding with the matching for the next time interval. This process proceeded sequentially from Day0-30 interval up to day 570-600 (last interval with switchers).

Since this was done without replacement, individuals who switched at a latter interval could be selected as a matched non-switcher. These individuals were treated as non-switchers for the rest of the analysis. Replacement was not done since it complicates the analysis by the need to calculate additional analytic weights.

1. **Bayesian modeling**

Bayesian longitudinal models as implemented in the `brms` package were used to model eGFR^5^ over time according to switching status. `brms` is a package that converts R commands into a Bayesian model that is implemented via Stan.

The longitudinal eGFR model adjusted for age, gender, insurance, race & ethnicity, smoking status, comorbidity status at follow-up start date, weight at follow-up start date, eGFR at follow-up start date, eGFR at switch eligibility date, and duration between starting TDF to follow-up start date. Time was expressed in B-splines with 4 degrees of freedom. Interaction terms between time and switching status, time and eGFR at follow-up start date, and switching status and eGFR at follow-up start date to introduce model flexibility and capture potential treatment heterogeneity. Random effect terms for person and pair id were also included. The model used the default 4 chains with a total of 10,000 iterations per chain and a thinning rate of 3. We used an adapt_delta of 0.9 to reduce divergent iterations.

After estimating the outcome model, we used the `marginaleffects` and the `tidybayes` package to obtain estimate marginal differences between switching and non-switching scenarios. To accomplish this, we predicted eGFR for based on data from observed switchers only with switch status set to switch as well as the predicted eGFR if the same people where assigned to remain on TDF (i.e., not switch). We used the predictions function with re_formula=NA function to calculate the eGFR for each scenario and the comparisons function with re_formula=NA to get the difference between the two scenarios. These two functions were combined with the ‘posteriordraws’ function so we get posterior predictions from the model. For interpretation, the results can be interpreted as analogous to average treatment effect among the treated since we only used data from people who switched from TDF to TAF. The posterior draws were then used to generate point estimates and credible intervals as well as supplemental measures like % of draws showing increased eGFR or region of practical equivalence.

1. **Sensitivity analysis: Adherence**

To address the issue of treating some switchers as non-switchers, we repeated the Bayesian modeling but dropped eGFR observations after treatment deviation. That is, we dropped observations of switchers after they switched back to TDF and we dropped observations of non-switchers if they switched to TDF.

1. **Sensitivity analysis: Weighted Bayesian model**

Since we dropped individuals after matching, there is a possibility of introducing selection bias. To assess the impact of this, we repeated the Bayesian model but included analytic weights for probability of having no follow-up eGFR data. This inverse probability of missingness weight was calculated using a logistic regression model which included age, gender, race & ethnicity, insurance, smoking status, hypertension status, weight at switch eligible date, eGFR at switch eligible date, and duration from TDF start to switch eligible date. Stabilized weights were used for the analysis.

1. **Sensitivity analysis: Multiple imputation with linear models**

The last sensitivity analysis used a frequentist framework with linear models instead of a Bayesian longitudinal model. In this approach, we calculated a linear model for each follow-up time point. Missing outcome data were handled using multiple imputation (m=50, max iterations=5) with chained equations via the `mice` package.^6,7^ The percent of individuals with missing at least one eGFR per time interval were as follows:

- Month 0.5 to <3: 37%
- Month 3 to <6: 34%
- Month 6 to <9: 46%
- Month 9 to <12: 55%
- Month 12 to <15: 61%
- Month 15 to <18: 67%
- Month 18 to 21: 72%

The imputation model used the same covariates as described in the imputation with missForest. To obtain the marginal difference between switching scenarios, we used the comparisons function from `marginaleffects` limited to data of observed switchers and used robust estimator (HC3) to obtain the confidence intervals. Results from the different imputations were pooled using Rubin’s rules.

1. **References cited**

1. Stekhoven DJ, Buhlmann P. MissForest--non-parametric missing value imputation for mixed-type data. *Bioinformatics*. Jan 1 2012;28(1):112-8. doi:10.1093/bioinformatics/btr597

2. Zhang Z, Li X, Wu X, Qiu H, Shi H, written on behalf of AMEB-DCTCG. Propensity score analysis for time-dependent exposure. *Ann Transl Med*. Mar 2020;8(5):246. doi:10.21037/atm.2020.01.33

3. *R survival package*. R Core Team; 2013.

4. *optmatch*. 2018. <https://github.com/markmfredrickson/optmatch>

5. Bürkner P-C. brms: An R Package for Bayesian Multilevel Models Using Stan. *Journal of Statistical Software*. 2017;80(1)doi:10.18637/jss.v080.i01

6. Van Buuren S, Groothuis-Oudshoorn K. mice: Multivariate imputation by chained equations in R. *Journal of Statistical Software*. 2011;45:1-67.

7. *_marginaleffects: Predictions, Comparisons, Slopes, Marginal Means, and Hypothesis Tests_.* 2023. <https://marginaleffects.com/>

1. **Sample R code**

########I. Single Imputation using missForest

#questions about code should be sent to Adovich Rivera, [ado.s at kp.org] or [adovichrivera2021 at u.northwestern.edu]

library(survival)

library(doParallel)

library(missForest)

o2_surv_wide_fin <- o2_surv_wide %>%

filter(Time_event_prep_end > 0) %>% #remove those who get censored for any reason before index date

mutate(Event_switch = ifelse(is.na(Time_switch),0,

ifelse(Time_switch<=Time_cens_prep_end,1,0)))

temp <- o2_surv_wide_fin %>%

mutate(htn_tdf_bl = ifelse(is.na(Time_htn), 0, ifelse(Time_htn<=0,1,0)),

dm_tdf_bl = ifelse(is.na(Time_dm), 0, ifelse(Time_dm<=0,1,0)),

dyslipid_tdf_bl = ifelse(is.na(Time_dyslipid), 0, ifelse(Time_dyslipid<=0,1,0))

) %>%

#select variables to include in imputation model

dplyr::select(age_bl, race_5cat, male_gender, ever_smoked_bl,

ins_comm, ins_gov, ins_oth, ins_comb,

index_yr, Time_index_dt, #time to eligibility

dm_tdf_bl, dyslipid_tdf_bl, htn_tdf_bl,

Time_event, Event_switch, Time_event_prep_end,

wt_kg_tdf_bl, bmi_tdf_bl, egfr_tdf_bl,

wt_kg_index_bl, bmi_index_bl, egfr_index_bl) %>%

mutate(index_yr=index_yr-2019) %>%

mutate(race_5cat = factor(race_5cat),

ins_comb = factor(ins_comb))

set.seed(12345)

registerDoParallel(cores=5)

xmis <- missForest(as.data.frame(temp), maxiter=10, ntree=100, parallelize='forests')

########II. Time-varying propensity score matching

####A. Set-up data – create a tmerge version of the data

temp <- o2_surv_wide_fin %>%

mutate(miss_wt_kg = ifelse(is.na(wt_kg_tdf_bl),1,0),

miss_egfr = ifelse(is.na(egfr_tdf_bl),1,0)) %>%

dplyr::select(mrn, age_bl, race_5cat, male_gender, index_yr,

wt_kg_index_bl_imp, bmi_index_bl_imp, egfr_index_bl_imp,

miss_wt_kg, miss_egfr, ever_smoked_bl,

ins_comm, ins_gov, ins_comb, medcen_bl,

Time_event_prep_end, Time_index_dt, Time_switch, Time_cens_prep_end, Event_switch) %>%

mutate(index_yr=index_yr-2019)

o2_surv_tm <- tmerge(temp, temp, id=mrn, endpt = event(Time_event_prep_end, Event_switch)) %>%

mutate(medcen_bl = ifelse(is.na(medcen_bl),'99',medcen_bl),

ins_comm = ifelse(is.na(ins_comm),0,ins_comm),

ins_gov = ifelse(is.na(ins_gov),0,ins_gov))

temp <- o2_surv_wide_fin %>% dplyr::select(mrn, Time_htn, Time_dm, Time_dyslipid)

o2_surv_tm <- tmerge(o2_surv_tm, temp, id=mrn,

htn=tdc(Time_htn), dm=tdc(Time_dm), dyslipid=tdc(Time_dyslipid))

temp <- o2_surv_wide_fin %>% dplyr::select(mrn) %>% distinct(.) %>%

mutate(id_num=row_number()) ##add an id_num variable

o2_surv_tm <- left_join(o2_surv_tm, temp,

by='mrn') %>%

ungroup()

####B. Estimate Cox model and cumulative hazard

PSmodCox <- coxph(Surv(tstart, tstop, Event_switch)~

age_bl + race_5cat + male_gender + ever_smoked_bl +

ins_comb + index_yr +

wt_kg_index_bl_imp + egfr_index_bl_imp + log(Time_index_dt) +

htn + dm + dyslipid, data=o2_surv_tm)

surObj <- survfit(PSmodCox, newdata=o2_surv_tm)

PScore <- data.frame(Time=surObj$time, surObj$cumhaz)

####C. Create long dataset for matching with cumulative hazard for each 30-day time interval

PScore_long <- PScore %>%

pivot_longer(cols=starts_with("X"),

values_to='cumhaz')

PScore_long <- PScore_long %>%

mutate(id_num=as.numeric(str_replace(name,"X","")))

dtScore <- merge(PScore_long,

o2_surv_tm %>%

dplyr::select(mrn, id_num, Time_switch, Event_switch, Time_cens_prep_end, Time_event_prep_end),

by='id_num')

dtScoreStrata <- dtScore %>%

filter(Time >= 0) %>%

mutate(TimeStrata = cut(Time, breaks=seq(0,1020, 30), include.lowest=T),

Cens_Strata = ifelse(Time < Time_cens_prep_end, 0, 1),

Switch_Strata = ifelse(Event_switch==0, 0,

ifelse(Time < Time_event_prep_end, 0, 1)))

dtScoreStrata_fin <- dtScoreStrata %>%

group_by(mrn, TimeStrata) %>%

slice(which.max(cumhaz)) #this selects the max cumulative hazard

#####D. Perform matching without replacement

library(optmatch)

dtFull <- dtScoreStrata_fin

DtMatched <- NULL

strata <- as.character(sort(unique(dtScoreStrata$TimeStrata)))

strataNo <- 34

#this does the matching for the first interval

dtStrata1 <- dtFull %>%

filter(TimeStrata==strata[[1]]) %>%

filter(Time < Time_cens_prep_end) #excludes people who are censored by start of Time strata -> should I revise to round up?

mahal.match1 <- pairmatch(

match_on(Switch_Strata ~ cumhaz,data=dtStrata1),

data=dtStrata1,

controls=4)

DTwithGrp1 <- cbind(dtStrata1, matches=mahal.match1)

dtMatched_fin <- DTwithGrp1[!is.na(DTwithGrp1$matches),]

exclude_fin <-dtMatched_fin$id_num #this marks individuals for exclusion in the matching

#this loops over the rest of the intervals

for(ii in 2:34){

print(ii)

#a. gets data of people in strata and remove if censored already

strata_txt <- strata[ii]

dtStrata2 <- dtFull[dtFull$TimeStrata==strata_txt,] %>%

filter(Time < Time_cens_prep_end) %>%

filter(!is.element(id_num, exclude_fin))

print(paste("switchers=",nrow(dtStrata2 %>% filter(Switch_Strata==1))))

#b. if at least one person is exposed within strata, do a match

if(sum(dtStrata2$Switch_Strata!=0)){

mahal.match <- pairmatch(

match_on(Switch_Strata ~ cumhaz,data=dtStrata2),

data=dtStrata2,

controls=4)

#this flags the matched pairs

DTwithGrp <- cbind(dtStrata2, matches=mahal.match)

print(paste0('matched=',nrow(DTwithGrp %>% filter(!is.na(matches) & Switch_Strata==1))))

#bring out matched and add to dtMatched_fin

dtMatched <- DTwithGrp[!is.na(DTwithGrp$matches),]

dtMatched_fin <- rbind(as.data.frame(dtMatched_fin),

as.data.frame(dtMatched))

#update exclude_fin

exclude_fin <- c(exclude_fin, dtMatched_fin$id_num)

} else {

next

}

}

########III. Main Analysis

library(brms)

library(marginaleffects)

####A. Calculating the Bayesian model

bmod <- brm(val_num ~ 0 + Intercept + age_bl + male_gender + ins_comb + race_5cat +

ever_smoked_bl + htn12_index + wt_kg_old_index_imp +

gfr_ct_bl + gfr_ave_bl_cen + egfr_old_index_imp + Time_tdf1_index +

bs(time10, df=4) + A +

bs(time10, df=4):A + A*gfr_ave_bl_cen + gfr_ave_bl_cen:bs(time10, df=4) +

(1|mrn) + (1|pair_id),

iter=10000,

control = list(adapt_delta = 0.9),

thin=3,

cores=4,

data=mod.data) #mod.data is the long format dataset including only matched switchers and non-switchers

####B. Calculating predicted eGFR

##Create the dataset for prediction

temp <- mod.data %>% ungroup() %>% filter(A==1) %>%

dplyr::select(mrn, pair_id, age_bl, male_gender, ins_comb, race_5cat, ever_smoked_bl, htn12_index,

gfr_ct_bl, gfr_ave_bl_cen, egfr_old_index_imp, wt_kg_old_index_imp, Time_tdf1_index) %>%

distinct(mrn, .keep_all=TRUE)

temp2 <- rbind(temp %>% mutate(A=0),

temp %>% mutate(A=1)) %>% dplyr::arrange(mrn) %>%

ungroup()

newdata <- rbind(temp2 %>% mutate(time10=1.5),

temp2 %>% mutate(time10=3*3),

temp2 %>% mutate(time10=3*6),

temp2 %>% mutate(time10=3*9),

temp2 %>% mutate(time10=3*12),

temp2 %>% mutate(time10=3*15),

temp2 %>% mutate(time10=3*18))

##Calculate the predicted eGFR for the two scnenarios

#baseline

bmod_blA0 <- predictions(bmod,

newdata=newdata %>% filter(A==0 & time10==1.5) %>% mutate(time10=0),

re_formula=NA) %>% posteriordraws()

#non-switching scenario

bmod_A0 <- predictions(bmod,

newdata=newdata %>% filter(A==0),

re_formula=NA) %>% posteriordraws()

#switching scenario

bmod_A1 <- predictions(bmod,

newdata=newdata %>% filter(A==1),

re_formula=NA) %>% posteriordraws()

####C. Difference in eGFR between switching scenarios

res1 <- comparisons(bmod,

variables=c("A"),

newdata = newdata %>% filter(time10==1.5),

re_formula=NA) %>% posteriordraws()

res.fin <- res1

times <- c(3*3, 3*6, 3*9, 3*12, 3*15, 3*18)

#loops over the time points

for(t in times){

print(t)

res2 <- comparisons(bmod,

variables=c("A"),

newdata = newdata %>% filter(time10==t),

re_formula=NA) %>% posteriordraws()

res.fin <- rbind(res.fin,res2)

}

########IV. Sensitivity Analysis: Weighted Bayesian models

####A. Calculating missingness weights

wt.num <- glm(include_fin ~ 1, 'binomial', mw.data)

wt.denom <- glm(include_fin ~ age_bl + male_gender + race_5cat + ins_comb +

ever_smoked_bl + htn12_index + wt_kg_old_index_imp + egfr_old_index_imp + Time_tdf1_index,

'binomial', mw.data)

mw.data$p.num <- predict(wt.num, newdata=mw.data, type='response')

mw.data$p.den <- predict(wt.denom, newdata=mw.data, type='response')

mw.data$wt.cc <- 1/mw.data$p.den

mw.data$wt.cc.stab <- mw.data$p.num/mw.data$p.den #stabilized weights

mod.data.wt <- mod.data %>%

left_join(.,

mw.data %>% dplyr::select(mrn, wt.cc, wt.cc.stab),

by='mrn')

####B. Calculating the Bayesian model

bmod.wt <- brm(val_num|weights(wt.cc) ~ 0 + Intercept + age_bl + male_gender + ins_comb + race_5cat +

ever_smoked_bl + htn12_index + wt_kg_old_index_imp +

gfr_ct_bl + gfr_ave_bl_cen + egfr_old_index_imp + Time_tdf1_index +

bs(time10, df=4) + A +

bs(time10, df=4):A + A*gfr_ave_bl_cen + gfr_ave_bl_cen:bs(time10, df=4) +

(1|mrn) + (1|pair_id),

iter=10000,

control = list(adapt_delta = 0.9),

thin=3,

cores=4,

data=mod.data.wt)

####C. Calculating predicted eGFR

temp <- mod.data.wt %>% ungroup() %>% filter(A==1) %>%

dplyr::select(mrn, pair_id, age_bl, male_gender, ins_comb, race_5cat, ever_smoked_bl, htn12_index,

gfr_ct_bl, gfr_ave_bl_cen, egfr_old_index_imp, wt_kg_old_index_imp, Time_tdf1_index, wt.cc) %>%

distinct(mrn, .keep_all=TRUE)

temp2 <- rbind(temp %>% mutate(A=0),

temp %>% mutate(A=1)) %>% dplyr::arrange(mrn) %>%

ungroup()

newdata <- rbind(temp2 %>% mutate(time10=1.5),

temp2 %>% mutate(time10=3*3),

temp2 %>% mutate(time10=3*6),

temp2 %>% mutate(time10=3*9),

temp2 %>% mutate(time10=3*12),

temp2 %>% mutate(time10=3*15),

temp2 %>% mutate(time10=3*18))

bmod.wt_blA0 <- predictions(bmod.wt,

wts='wt.cc',

newdata=newdata %>% filter(A==0 & time10==1.5) %>% mutate(time10=0),

re_formula=NA) %>% posteriordraws()

bmod.wt_A0 <- predictions(bmod.wt,

wts='wt.cc',

newdata=newdata %>% filter(A==0),

re_formula=NA) %>% posteriordraws()

bmod.wt_A1 <- predictions(bmod.wt,

wts='wt.cc',

newdata=newdata %>% filter(A==1),

re_formula=NA) %>% posteriordraws()

####D. Difference in eGFR between switching scenarios

res1 <- comparisons(bmod.wt,

wts='wt.cc',

variables=c("A"),

newdata = newdata %>% filter(time10==1.5),

re_formula=NA) %>% posteriordraws()

res.fin.wt <- res1

times <- c(3*3, 3*6, 3*9, 3*12, 3*15, 3*18)

for(t in times){

print(t)

res2 <- comparisons(bmod.wt,

wts='wt.cc',

variables=c("A"),

newdata = newdata %>% filter(time10==t),

re_formula=NA) %>% posteriordraws()

res.fin.wt <- rbind(res.fin.wt,res2)

}

########V. Sensitivity Analysis: Linear models

####A. Imputing missing outcome data

library(mice)

#set-up wide data with eGFR for each follow-up time point

mod.data.marg <- wide_df %>%

#egfr

left_join(., egfr_bl,by='mrn') %>%

left_join(., egfr_1, by='mrn') %>%

left_join(., egfr_2, by='mrn') %>%

left_join(., egfr_3, by='mrn') %>%

left_join(., egfr_4, by='mrn') %>%

left_join(., egfr_5, by='mrn') %>%

left_join(., egfr_6, by='mrn') %>%

left_join(., egfr_7, by='mrn')

mi.data <- mod.data.marg %>%

dplyr::select(mrn, pair_id, age_bl, male_gender, ins_gov, ins_comm, race_5cat,

gfr_ct_bl, gfr_ave_bl, egfr_old_index_imp, wt_kg_old_index_imp,

dm_index, htn12_index, dyslipid_index,

A, gfr1, gfr2, gfr3, gfr4, gfr5, gfr6, gfr7) %>% ungroup()

#set-up imputation models

m = 50

mi0 <- mice(mi.data,maxit=1,m=1)

meth <- mi0$method

pred <- mi0$predictorMatrix

pred[,c('mrn')] <- 0

pred[c('mrn'),] <- 0

#conduct multiple imputation

mice.egfr <- mice(mi.data, method=meth, predictorMatrix = pred,

m=m, maxit=5, seed=12345)

####B. Calculating the linear model

##obtain imputed datasets in a list format

dat_long <- complete(mice.egfr,"all")

##set-up function to calculate the ATT given data and a formula

get_comp_res <- function(dat,var,modform=formula){

require(broom)

require(marginaleffects)

# var='gfr2'

# modform = formula

# dat <- dat_long[[1]]

dat$y <- dat[[var]]

mod <- lm(formula, dat)

comp <- comparisons(mod,

var="A",

newdata=dat %>% filter(A==1),

vcov="HC3")

res <- tidy(comp) %>% dplyr::select(term, estimate, std.error, conf.low, conf.high)

return(res)

}

####C. Calculating the difference in eGFR between switching scenarios

#set-up outcome formula

formula <- as.formula('y ~ A*gfr_ave_bl +

age_bl + male_gender + ins_gov + ins_comm + race_5cat +

htn12_index + dyslipid_index +

gfr_ct_bl + egfr_old_index_imp')

#calculate ATT results for follow-up time 1.5

comp_list <- lapply(X=seq(1,50),FUN=

function(X){

dat = dat_long[[X]]

res <- get_comp_res(dat,var='gfr1') %>%

mutate(m=X)

return(res)

})

mi_gfr1_res <- do.call(rbind, comp_list) %>% mutate(time=1.5) #can repeat this for the other follow-up time points

####D. Pooling results across imputations

#set-up function to get pooled results from imputations

get_pooled_res <- function(pooled_res,m=50){

#pooled_res = mi_wt_p1_res

t = pooled_res$time[[1]]

theta = mean(pooled_res$estimate)

v_w = mean(pooled_res$std.error^2)

v_b = sum((pooled_res$estimate-theta)^2)/(m-1)

v_tot = v_w + v_b + v_b/m

se = sqrt(v_tot)

theta.ll = theta - 1.96*se

theta.ul = theta + 1.96*se

res <- data.frame(gfr_time=t, theta=theta, se=se,

theta.ll=theta.ll, theta.ul=theta.ul)

return(res)

}

#get results

get_pooled_res(mi_gfr1_res)
