# Supplementary material for: Changes in Glomerular Filtration Rate After Switching From Tenofovir Disoproxil Fumarate to Tenofovir Alafenamide Fumarate for Human Immunodeficiency Virus Preexposure Prophylaxis
Source: Open Forum Infect Dis. 2023 Dec 29;11(2):ofad695. doi: 10.1093/ofid/ofad695 (PMC10863550; doi:10.1093/ofid/ofad695)
Supplement: ofad695_Supplementary_Data [file ofad695_supplementary_data.zip › prep o2 - eGFR and TAF swtiching - SFile 2 - results.docx]

**Supplement File 2: Supplemental Results**

**Changes in Glomerular Filtration Rate after Switching from Tenofovir Disoproxil Fumarate to Tenofovir Alafenamide Fumarate for HIV Pre-exposure Prophylaxis**

Adovich S. Rivera, MD, PhD^1^

Katherine Pak, MS^1^

Matthew Mefford, PhD^1^

Rulin C. Hechter, MD, PhD^1.2^

^1^ – Department of Research and Evaluation, Kaiser Permanente Southern California

^2^ – Health Systems Science, Kaiser Permanente Bernard J. Tyson School of Medicine

*List of Supplemental Tables*

Table S1. Summary Characteristics of Eligible Individuals At Switch Eligible Date (n = 5,246) , Kaiser Permanente Southern California, October 2019 – June 2022.

Table S2. Proportion of Direction of Association and Region of Practical Equivalence for Difference in estimated Glomerular Filtration Rate between Switching Scenarios, Kaiser Permanente Southern California, October 2019 – June 2022.

*List of Supplemental Figures*

Figure S1. Creation of Matched Analytic Cohort from Electronic Health Records of Kaiser Permanente Southern California, October 2019 – June 2022.

Figure S2. Aggregated estimated glomerular filtration rate over time in matched sample, Kaiser Permanente Southern California, October 2019 – June 2022.

Figure S3. Estimated Difference in estimated Glomerular Filtration Rate (eGFR) Between Switching and Non-Switching Scenarios based on model restricted to adherent data, Kaiser Permanente Southern California, October 2019 – June 2022.

Figure S4. Estimated Difference in estimated Glomerular Filtration Rate (eGFR) Between Switching and Non-Switching Scenarios based on model with inverse missingness weights, Kaiser Permanente Southern California, October 2019 – June 2022

Table S1. Summary Characteristics of Eligible Individuals At Switch Eligible Date (n = 5,246) , Kaiser Permanente Southern California, October 2019 – June 2022.

| **Variables** | **Statistic** |
| --- | --- |
| Age, years, mean (SD) | 37.92 (11.04) |
| Race and ethnicity (%) |  |
| Asian, non-Hispanic | 549 (10.5) |
| Black, non-Hispanic | 275 ( 5.2) |
| Hispanic | 1848 (35.2) |
| White, non-Hispanic | 2,096 (40.0) |
| Other, non-Hispanic | 478 (9.1) |
| Male (%) | 5,146 (98.1) |
| Insurance type (%) |  |
| Commercial | 4,209 (80.3) |
| Medicaid | 241 ( 4.6) |
| Medicare | 77 ( 1.5) |
| Other | 716 (13.7) |
| Ever smoked (%) | 1,396 (26.6) |
| Diabetes (%) | 257 (4.9) |
| Dyslipidemia (%) | 795 (15.2) |
| Hypertension (%) | 1,304 (24.9) |
| Weight, kg, mean (SD) | 74.34 (17.13) |
| eGFR, mL/kg/m^2^, mean (SD) | 101.51 (16.43) |
| Time to meeting switch eligibility, days, mean (SD) | 477.02 (414.84) |
| Switched to TAF during follow-up (%) | 125 (2.4) |

Note: Other race and ethnicity include multiracial, Native American/Alaskan, Pacific Islander, and all other types of responses not reported in the figure. eGFR – estimated glomerular filtration rate calculated using the CKD 2021 EPI Creatinine equation, TAF – tenofovir alafenamide fumarate

Table S2. Proportion of Direction of Association and Region of Practical Equivalence for Difference in estimated Glomerular Filtration Rate between Switching Scenarios, Kaiser Permanente Southern California, October 2019 – June 2022.

| **Follow-up Time**  **(months)** | **Draws showing higher eGFR with switch to TAF (%)** | **% Draws outside of ROPE (±2%) (%)** |
| --- | --- | --- |
| 0.5 | 52.3 | 40.8 |
| 3 | 94.0 | 50.8 |
| 6 | 86.6 | 29.5 |
| 9 | 83.5 | 29.7 |
| 12 | 95.5 | 58.0 |
| 15 | 91.7 | 49.6 |
| 18 | 22.0 | 53.9 |

Abbreviations: eGFR - estimated Glomerular Filtration based on the the CKD 2021 EPI Creatinine equation, ROPE – region of practical equivalence

Figure S1. Creation of Matched Analytic Cohort from Electronic Health Records of Kaiser Permanente Southern California, October 2019 – June 2022.


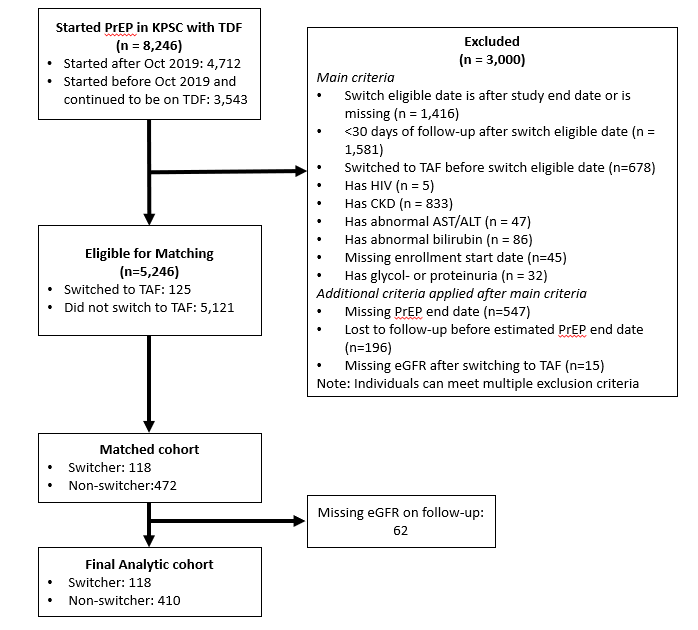


Abbreviations: ALT – alanine transaminase, AST – aspartate transaminase, CKD – chronic kidney disease, eGFR – estimated glomerular filtration rate, HIV – human immunodeficiency virus, PrEP – pre-exposure prophylaxis, TAF – tenofovir alafenamide fumarate, TDF – tenofovir disoproxil fumarate

Figure S2. Aggregated estimated glomerular filtration rate over time in matched sample, Kaiser Permanente Southern California, October 2019 – June 2022.
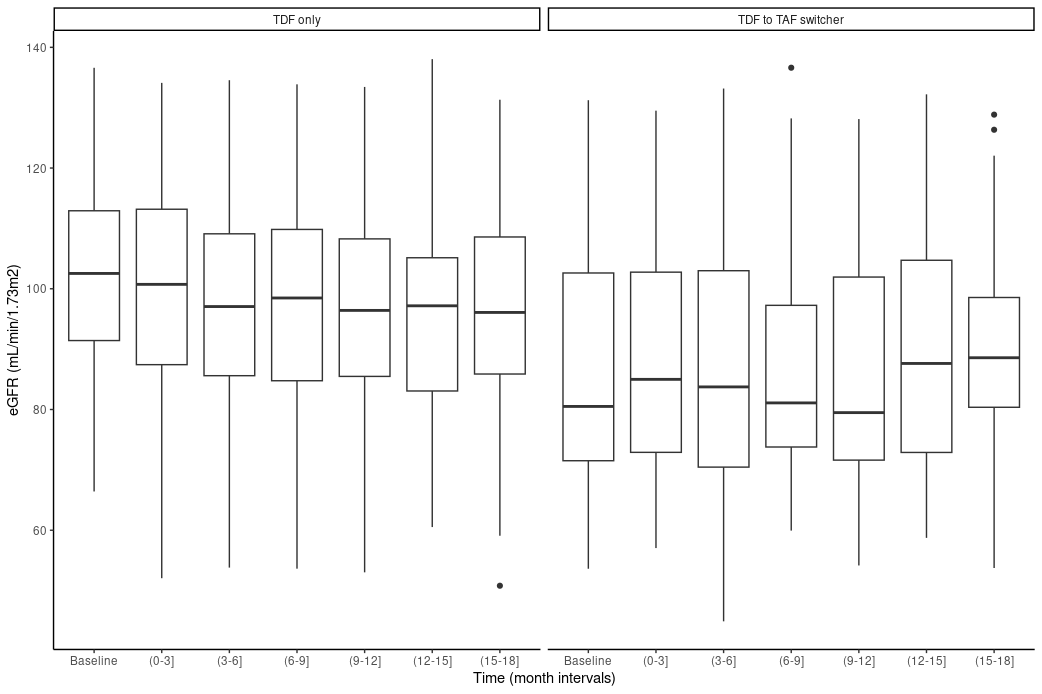


Abbreviations: eGFR – estimated glomerular filtration based on the CKD 2021 EPI Creatinine equation, TAF – tenofovir alafenamide fumarate, TDF – tenofovir disoproxil fumarate

Figure S3. Estimated Difference in estimated Glomerular Filtration Rate (eGFR) Between Switching and Non-Switching Scenarios based on model restricted to adherent data, Kaiser Permanente Southern California, October 2019 – June 2022.
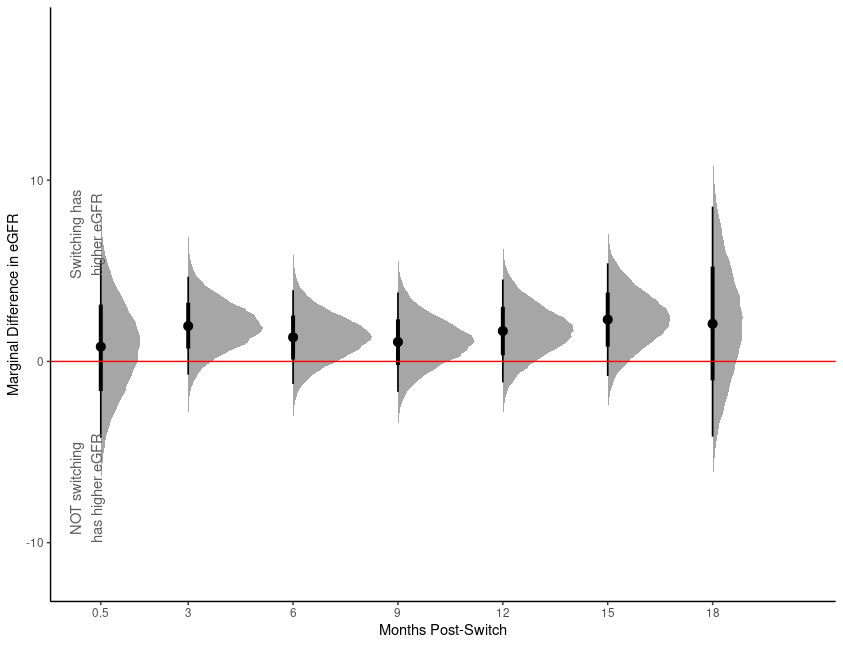


Note: eGFR – estimated glomerular filtration rate based on the CKD 2021 EPI Creatinine equation. Grey density curve show distribution of estimated differences. Dot is median of estimated differences. Thick lines show 66% credible interval and thin lines show 95% credible interval. Red line is threshold for no difference.

Figure S4. Estimated Difference in estimated Glomerular Filtration Rate (eGFR) Between Switching and Non-Switching Scenarios based on model with inverse missingness weights, Kaiser Permanente Southern California, October 2019 – June 2022


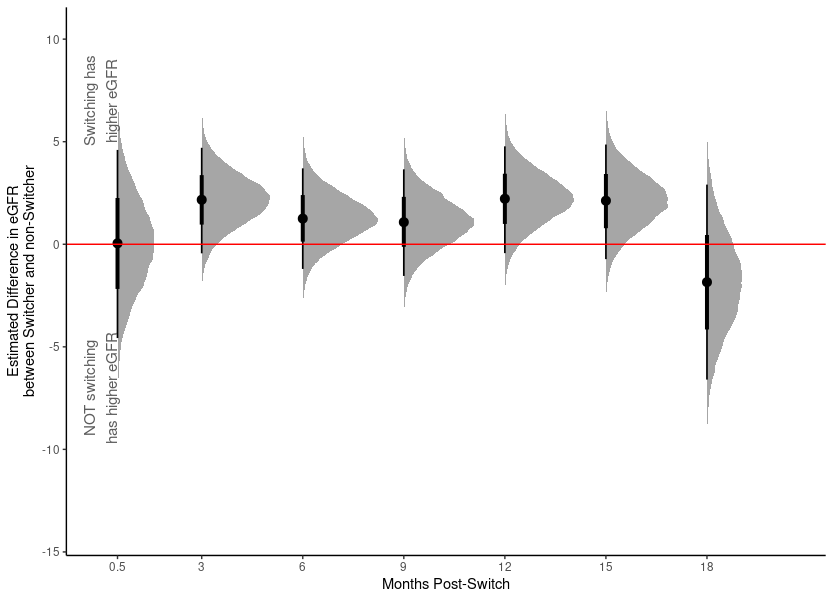


Note: eGFR – estimated glomerular filtration rate calculated using the CKD 2021 EPI Creatinine equation. Grey density curve show distribution of estimated differences. Dot is median of estimated differences. Thick lines show 66% confidence interval and thin lines show 95% confidence interval. Red line is threshold for no difference.
